# Using a Convolutional Siamese Network for Image-Based Plant Species Identification with Small Datasets

**DOI:** 10.3390/biomimetics5010008

**Published:** 2020-03-01

**Authors:** Geovanni Figueroa-Mata, Erick Mata-Montero

**Affiliations:** 1 School of Mathematics, Costa Rica Institute of Technology, calle 15, avenida 14, Cartago 30101, Costa Rica; 2 School of Computing, Costa Rica Institute of Technology, calle 15, avenida 14, Cartago 30101, Costa Rica; emata@itcr.ac.cr

**Keywords:** automated species identification, k-shot learning, similarity function, convolutional siamese network

## Abstract

The application of deep learning techniques may prove difficult when datasets are small. Recently, techniques such as one-shot learning, few-shot learning, and Siamese networks have been proposed to address this problem. In this paper, we propose the use a convolutional Siamese network (CSN) that learns a similarity metric that discriminates between plant species based on images of leaves. Once the CSN has learned the similarity function, its discriminatory power is generalized to classify not just new pictures of the species used during training but also entirely new species for which only a few images are available. This is achieved by exposing the network to pairs of similar and dissimilar observations and minimizing the Euclidean distance between similar pairs while simultaneously maximizing it between dissimilar pairs. We conducted experiments to study two different scenarios. In the first one, the CSN was trained and validated with datasets that comprise 5, 10, 15, 20, 25, and 30 pictures per species, extracted from the well-known Flavia dataset. Then, the trained model was tested with another dataset composed of 320 images (10 images per species) also from Flavia. The obtained accuracy was compared with the results of feeding the same training, validation, and testing datasets to a convolutional neural network (CNN) in order to determine if there is a threshold value *t* for dataset size that defines the intervals for which either the CSN or the CNN has better accuracy. In the second studied scenario, the accuracy of both the CSN and the CNN—both trained and validated with the same datasets extracted from Flavia—were compared when tested on a set of images of leaves of 20 Costa Rican tree species that are not represented in Flavia.

## 1. Introduction

Deep learning is an approach to machine learning that in recent years has experienced tremendous growth and success, largely as a result of the availability of more powerful computers, larger datasets, and new techniques to train artificial neural networks.

Artificial neural networks (ANNs), as their name suggests, are loosely inspired by the animal nervous system. An ANN is structured as a weighted directed graph in which nodes represent neurons, arcs represent connections that carry information from one neuron to another, and weights denote the relative importance of the corresponding arc. At a more general level, ANNs are structured as a linear sequence of layers, each of which receives inputs (arcs) from the previous layer, performs nonlinear computations in its nodes, and relays the results to the neurons in the next layer.

Convolutional neural networks (CNNs) are one type of ANN used in deep learning to process large amounts of data. They have a particular multilayer architecture. In each layer, convolutional operations are run interspersed with nonlinear activation functions and pooling operations (pooling layer) which enable them to learn nonlinear representations. They have been used as state-of-the-art classifiers that have achieved outstanding performance on different tasks, such as image classification and speech recognition, among others. One very powerful characteristic of all deep learning architectures—CNNs included—is that they use feature learning techniques, that is, they automatically discover the representations needed for feature detection or classification from the input data. Feature learning techniques can be roughly classified as supervised or unsupervised. We limit this brief introduction to the type of learning technique used in this work, namely, supervised feature learning with CNNs.

Supervised learning uses labeled input data to discover (learn) features. During the training phase, the CNN is fed with a dataset (training set) in order to tune its parameters. For this, a loss function that measures the difference between the target value (label) and the value predicted by the network is minimized. Additionally, the network is evaluated with another dataset (validation set) in order to monitor its performance and prevent overfitting.

The training set is fed into the convolutional neural network in batches. When the entire training set has passed through the neural network once, we say that an epoch has been completed. In each epoch, the network parameters are tuned until the desired accuracy is reached or little improvement in accuracy is detected.

In spite of the enormous success of deep learning, accruing enough data to increase the accuracy of the models is often not possible or difficult. For example—in the context of plant species identification—collecting, processing, and labeling (identifying) samples to turn them into supervised data can be a very expensive, time-consuming, error-prone, and even unfeasible task. This is exacerbated because—for quality assurance reasons—expert taxonomists should be involved. Furthermore, collecting often needs to be performed in places that are difficult to access such as tropical forests.

In contrast, humans have a great ability to recognize new patterns and learn from few examples. For instance, a person only needs to see one elephant to acquire the concept and will most likely be able to discriminate future elephants from other animals such as giraffes and zebras. How is few-shot learning possible? A number of authors have suggested that new concepts are almost never learned in a vacuum. Past experience with other concepts in a domain can support the rapid learning of novel concepts [1].

Several techniques have been proposed to address the training of models when little supervised data is available, among them, zero-shot [2], one-shot [3,4,5], few-shot or *k*-shot learning [6,7], and Siamese networks [8,9].

This work aims at studying the potential of convolutional Siamese networks (CSN) for plant species identification based solely on small datasets of leaf images. More specifically, we study two scenarios with the following associated goals:

1. **Finding a threshold value (Scenario 1)**: Determine if there is a threshold value *t* for dataset size that defines the intervals for which either a CSN or a CNN architecture has better accuracy. Our hypothesis is that for “small” values of *t* a CSN is preferable.
2. **Scalability with small datasets (Scenario 2)**: Compare the accuracy of a CSN and a CNN—both trained and validated with the same datasets extracted from Flavia—when tested on a small set of images of leaves of 20 Costa Rican tree species that are not represented in Flavia.

Our approach learns image representations via a supervised metric-based technique that uses a convolutional Siamese network and then it reuses that network’s features for k-shot learning (explained in Section 2.2) without any retraining. A convolutional Siamese network learns a similarity function from pairs of images. It does not attempt to classify images; it only discriminates if the two input images belong to the same class (species). Once the convolutional Siamese network has been tuned, its discriminatory power can be generalized to classify not only new images of the classes used during the training phase but also images of species unseen before in the training phase.

In our experiments, we restrict our attention to plant species identification based on small datasets of leaf images. Even though datasets of leaf images have become richer in terms of number of images per species and number of species, the number of images per species is typically distributed unevenly across species, which means that there will always be classes (species) that have very few samples for training purposes. Additionally, for certain regions of the planet—such as the Neotropic—where plant biodiversity is rich but image datasets are scarce, it is critical to be able to cope with small datasets. Conducting inventories of plant biodiversity efficiently and accurately is indispensable to monitor biodiversity trends and support biodiversity conservation measures. To our knowledge, research on automated identification of organisms using deep learning, although an active research area ([10,11,12,13,14,15]), has not addressed the issue when only small datasets are available.

For this work, we employ a convolutional Siamese network that

1. learns generic image features useful for making predictions about unknown class distributions even when very few examples from these new distributions are available;
2. is easily and quickly trainable by using standard optimization techniques on pairs sampled from the dataset; and
3. provides an alternative approach to deep learning that does not require large amounts of labeled samples.

This paper is organized as follows. Section 2 briefly presents related work, particularly on Siamese networks, *k*-shot *n*-way learning, and plant species identification using CNNs. Section 3 presents the methodology followed in the experiments. Section 4 discusses the results obtained in each experiment. Finally, we close in Section 5 with conclusions and future work.

## 2. Related Work

### 2.1. Siamese Networks

Siamese networks were first introduced in the 1990s. In 1993 they were used by Baldi et al. [8] for fingerprint recognition and by Bromley et al. [9] to solve a problem of signature verification. Convolutional Siamese networks (CSN) are a class of neural network architecture that contains two identical convolutional neural networks (CNN) that work in tandem to determine similarity between two inputs. Each twin CNN has the same configuration with the same parameters and shared weights. During the training phase, parameter updating is mirrored across both subnetworks. This framework has been successfully used for face verification [16], dimensionality reduction [17], comparing image patches [18], finding similar images [19], software defect prediction [20], and image recognition [5], among other applications.

### 2.2. k-Shot n-Way Learning

The general setting for a *k*-shot (a.k.a. few-shot) *n*-way learning scenario is the following:

- A model is given a query sample belonging to a new, previously unseen class.
- It is also given a support set, *S*, consisting of *k* examples each from *n* different unseen classes.
- The algorithm determines to which of *n* classes the query sample belongs.

In [5], Koch et al. propose a method for one-shot classification based on a convolutional Siamese network that learns a similarity function to discriminate between two input images. Then, they generalize the predictive power of the network not just to new data but to entirely new classes from unknown distributions.

### 2.3. Deep Learning Applied to Plant Identification

One way to understand the evolution of algorithms for the automatic identification of plants based on images and the state-of-the-art in recent years is through the results obtained in the global competition called PlantCLEF.

The PlantCLEF challenge is part of a larger challenge called LifeCLEF [21]. LifeCLEF has been around since 2011. Its goal is to improve the state-of-the-art in image-based identifications of organisms through a challenge in which scientists compete by using predefined image datasets of plants, birds, and fish. The PlantCLEF challenge includes not only digitized images of leaves but also of other components such as fruits, stems, and flowers.

**Figure 1:**
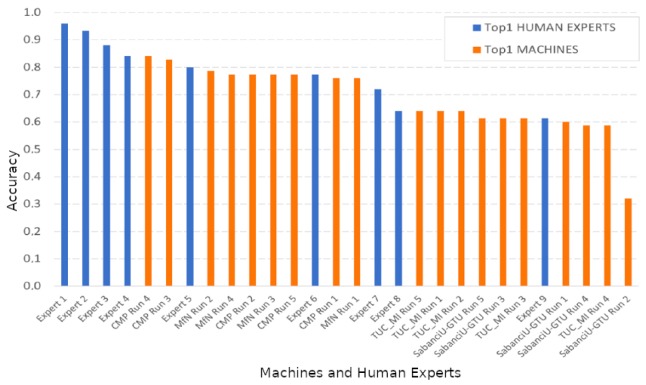
Identification performance achieved by the competing systems and participating human experts in PlantCLEF 2018. Figure taken from [12].

The results achieved in PLantCLEF since 2011 are indicative of what has happened with the algorithms to identify plants automatically from photos:

- The number of species (classes) in the datasets has grown from 71 in 2011 to 10,000 in 2018 and the number of photos from 5436 to more than 1,000,000.
- The highest top-1 accuracy achieved by the winning algorithm has grown from 0.5 to 0.867 in 2018. This is even more remarkable if we consider the amount of species that was used in 2018.
- From 2016 on, all the algorithms that competed used deep learning and CNNs, which demonstrates the supremacy of this approach over the traditional ones based on the extraction of predefined morphometric characteristics.
- In 2018 the accuracy of human experts and the best algorithms that competed was compared [12]. Figure 1 shows the results. Although experts are still better in general, the gap has been drastically reduced.

It is therefore clear that the deep learning algorithm’s performance—when large amounts of supervised data are at hand—has been so successful that it is now comparable to the performance of human experts. Dealing with few labeled data is still a challenge if we aim at achieving those accuracy levels.

In the traditional supervised learning classification approach, the input image to be classified is fed to a convolutional network, which produces an output in the form of a probability distribution of all the classes; the highest probability corresponds to the predicted class. This approach has two disadvantages. First, it needs a large amount of images for each class for the supervised training phase of the model [22,23]. Secondly, if the model was trained for *n* classes, then it cannot be tested on any other class [5,6].

## 3. Methodology

This section describes the experiments carried out in this research. First, Section 3.1 briefly characterizes the two datasets employed, namely, Flavia and CRleaves. Then, Section 3.2 presents a preliminary discussion about the criteria used to choose the architectures to be compared. Section 3.3 defines the CNN model used for both scenarios. This CNN is to be compared with a CSN, each of whose twin subnetworks uses this same CNN architecture. Section 3.4 presents details of the CSN, including its loss function, training approach, and plant species classification strategy. Finally, Section 3.5 describes the experiments conducted for the two studied scenarios, which were defined in Section 1. In Scenario 1 we want to find a threshold value *t* such that for training datasets of size less than or equal to *t*, the CSN may have better accuracy than the CNN. In Scenario 2 we want to assess the scalability of both the CSN and the CNN with respect to a small dataset that comprises 20 unseen tree species from CRleaves.

### 3.1. Datasets

**Figure 2:**
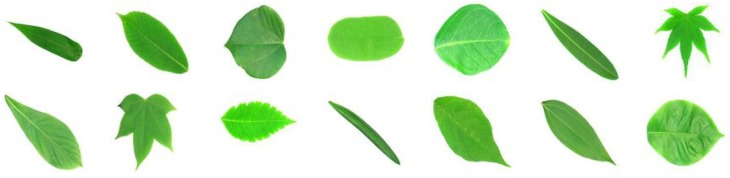
Some images of the Flavia dataset.

To train both the CSN and the CNN, we use the well-known Flavia dataset [24], which comprises 1907 images of leaves corresponding to 32 plant species (http://flavia.sourceforge.net/). Each image has a resolution of 1600×1200 pixels with a white background and the number of images by species is between 50 and 77. Figure 2 shows a random sample taken from the Flavia dataset.

**Figure 3:**
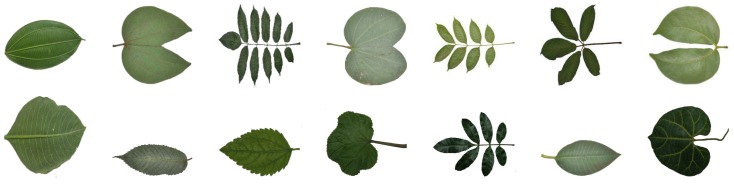
Some images of the CRleaves dataset.

We also use 20 randomly selected species from the CRleaves dataset created by Carranza et al. [25] to perform few-shot learning experiments with classes unseen during the training phase (second experiment). CRleaves includes 7262 images of leaves of 255 native species of Costa Rican trees. The number of images per species is between 4 and 36. Each image has a varying resolution and noisy background. Figure 3 shows a random sample taken from the CRleaves dataset.

Most of the images of the CRleaves dataset have a dark background with some noise; so, it was necessary to clean them. Then they are resized to 224 × 224 pixels and converted to grayscale.

It has been pointed out in [26] that certain biases may be introduced when datasets for training and testing are not selected rigorously. In particular, the Same-Specimen-Picture Bias (SSPB), described by Carranza et al. [26], is often inadvertently introduced because images of the same individual (even if these images are different) produce, in general, better accuracy results. This can only be avoided if the used datasets contain metadata that identifies individuals uniquely (not just the species name). Because Flavia does not include this metadata, it is not possible to avoid the SSPB. Nevertheless, this is not relevant in this work because our main interest is to assess the relative accuracy of the CSN model with respect to a CNN model, and they are both being affected by the SSPB.

### 3.2. Preliminary Discussion

We are looking for deep learning architectures that achieve good accuracy in spite of having small datasets. So, the starting point is a small dataset, a classification problem to solve, and a CNN that has size *n* (*n* parameters) but has low accuracy with the small dataset, although that accuracy could improve with larger datasets. This is a very realistic scenario in many cases, particularly, when a large number of classes (e.g., species of plants) have a small number of samples in each class.

Under those assumptions, it is not really relevant if, in the comparison, the CNN has roughly the same number of parameters as the CSN or not. As a matter of fact, if we have a CSN with 2n parameters (i.e., it uses two CNNs of size *n*) and compare it with a CNN of roughly 2n parameters instead of the original CNN with *n* parameters, the comparison may seem more fair. Nevertheless, that would most likely negatively affect the CNN as it has been observed [22] that CNNs with many parameters tend to perform well only with very large datasets. Therefore, we chose to compare a relatively “lean” CNN which has been successful in similar classification problems [5,6,16,17] with a CSN based on that architecture but, by definition of CSN, has roughly twice as many parameters.

The CNN architecture we used as a basis for the comparison in this research has roughly 503,000 parameters. Consequently, the CSN has roughly 1,000,006 parameters. In deep learning research these are not considered large (very deep) architectures. For example, a model such as MobileNet, which is one of the smallest in current research, has 4,253,864 parameters and Inception v3, which has been very successful but requires large training datasets, has 23,851,784 parameters [27]. It would be interesting in future work to confirm the hypothesis that those larger CNNs would perform even worse with the small datasets used in this research.

### 3.3. Convolutional Neural Network Model

**Figure 4:**
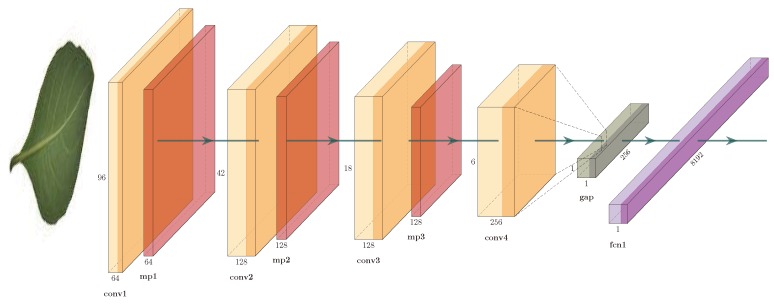
Convolutional architecture: convolutional neural networks (CNN).

We use a CNN similar to the one described in [5,16,17]. Figure 4 shows the architecture. It consists of three convolutional blocks: a convolutional layer with 32 filters of 11×11, a ReLU activation function, and a maxpooling layer; a convolutional layer with 64 filters of 8×8, a ReLU activation function, and a maxpooling layer; and a convolutional layer with 128 filters of 5×5 and a ReLU activation function. The units of this convolutional layer are flattened into a single vector using a global average-pooling (GAP) layer; this vector is connected to a fully-connected layer (FCN) with 1024 neurons, a ReLU activation function, and to a softmax layer.

### 3.4. Convolutional Siamese Netwok Model

As indicated in Section 2.1, convolutional Siamese networks are a class of CNN-based architecture that usually contains two identical CNNs. The twin CNNs have the same configuration with the same parameters and shared weights. The CNN model that we use to build our CSN is the one shown in Figure 4. Two copies of this subnetwork are joined by a loss function at the top, which computes a similarity metric that calculates the Euclidean distance between the feature vectors extracted by each subnetwork.

**Figure 5:**
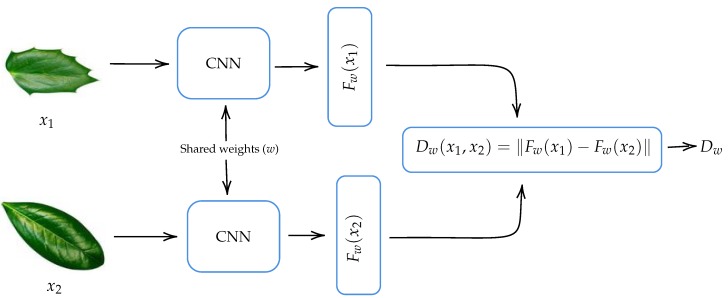
Diagram of the convolutional Siamese network (CSN).

Figure 5 shows a diagram of the convolutional Siamese network used. Here, $x_{1}$ and $x_{2}$ are the input images to our convolutional Siamese network, *w* represents a shared parameter vector, which is tuned during training phase, and $F_{w}\left(x_{1}\right)$ and $F_{w}\left(x_{2}\right)$ are the features vectors extracted by each of the convolutional neural networks that the Siamese network comprises. The output of the convolutional Siamese network $D_{w}=∥F_{w}\left(x_{1}\right)-F_{w}\left(x_{2}\right)∥$ measures the similarity between the feature vectors. Our hypothesis is that, on one hand, two images of leaves of the same species will have a similar feature vectors and therefore their distance is close to zero. On the other hand, two images of leaves of different species will have more different feature vectors and therefore their distance will be larger.

The similarity between the feature vectors $F_{w}\left(x_{1}\right)$ and $F_{w}\left(x_{2}\right)$ of input images $x_{1}$ and $x_{2}$ can be measured by distance metrics such as those induced by the norms $L_{1}$, $L_{2}$ and $L_{\infty }$ or with similarity function such as cosine similarity. In our case, we chose Euclidean distance, because it is widely used [16,19,20,28] and with it we got the best performance in preliminary tests.

#### 3.4.1. Loss Function Used for CSN Training

If $x_{1}$ and $x_{2}$ are a pair of input images, *w* represents shared parameter vector, and the mapping of $x_{1}$ and $x_{2}$ in the feature space is represented by $F_{w}\left(x_{1}\right)$ and $F_{w}\left(x_{2}\right)$, then the convolutional Siamese network can be considered as a measure function that measures the similarity between $x_{1}$ and $x_{2}$, by calculating the Euclidean distance between the feature vectors. This learned similarity measure function is defined as:

(1) $$D_{w}\left(x_{1},x_{2}\right)={∥F_{w}\left(x_{1}\right)-F_{w}\left(x_{2}\right)∥}_{2}$$

During the training phase of the convolutional Siamese network we use the contrastive loss function introduced by Chopra et al. in [16,17], which is defined as follows:

(2) $$L\left(w,y,x_{1},x_{2}\right)=\frac{y}{2}D_{w}{\left(x_{1},x_{2}\right)}^{2}+\frac{1-y}{2}{\left(\max\left\{0,m-D_{w}\left(x_{1},x_{2}\right)\right\}\right)}^{2}$$

where m>0 is a constant called a margin and *y* is a binary label assigned to the pair of input images $x_{1}$ and $x_{2}$, so that y=1 if the images belong to the same species and y=0 otherwise.

Note that if the images belong to the same species (y=1) their distance contributes to the loss function, while if they belong to different species (y=0), only those whose distance is less than or equal to *m* contribute. Therefore, minimizing $L(w,y,x_{1},x_{2})$ with respect to *w* would result in a small value of $D_{w}\left(x_{1},x_{2}\right)$ for images of the same species and a large value of $D_{w}\left(x_{1},x_{2}\right)$ for images of different species.

The value of *m* must be chosen experimentally and depends on the domain of application.

#### 3.4.2. Training the CSN Model

We initially extract at random and without replacement 10 images of each species, this set *P* of images is used to test our models and it does not change of experiment to experiment. This guarantees that the testing images will not be used in the training and validation phases. The remainder of the images are divided into two sets, one for training called *T* and the other for validation called *V*, such that T∩V=∅. These sets are built in a proportion of 80% to 20%, respectively, for the experiments.

An experiment $E_{n}$ is composed of a training set $T_{n}$ (taken from the set *T*), a validation set $V_{n}$ (taken from the set *V*), and the initially selected testing set *P*. For experiment $E_{n}$ we randomly selected *n* images of each species, such that 80% of them are for training and 20% for validation. For example, in the experiment $E_{10}$, 10 images per species are used, of these 8 are for training and 2 are for validation.

**Figure 6:**
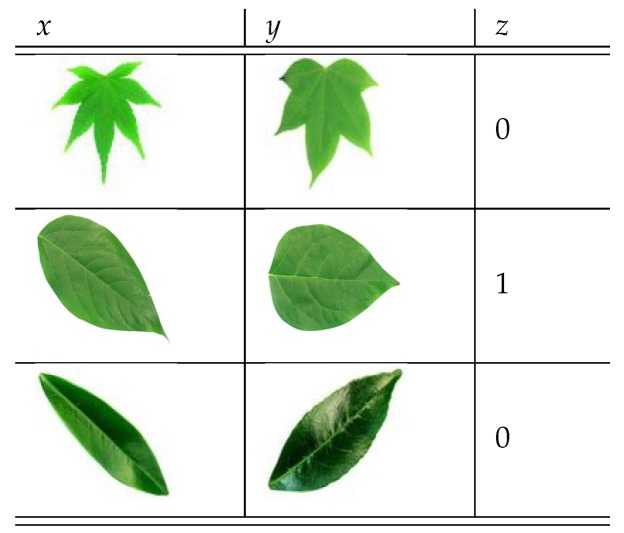
Example of 3-tuples used for the CSN training.

To train the CSN model we use batches of 3-tuples (x,y,z), where *x* and *y* are input images, and *z* is the corresponding label. If the images *x* and *y* belong to the same species z=1, otherwise z=0. Figure 6 shows an example of a batch of 3-tuples.

Algorithm 1 shows how the batches of 3-tuples are built, where, batch_size is the size of the batch, $T=\{C_{1},C_{2},\ldots ,C_{n}\}$ is the training set, $|C_{k}|$ is the number of images of the class $C_{k}$, and $T\left[C_{k}\right]\left[i\right]$ is the i-th image of the class $C_{k}$.

The images *x* and *y* of the 3-tuple are chosen at random and we use a probability of p=0.3 to decide if the images will belong to the same species.

**Figure 7:**
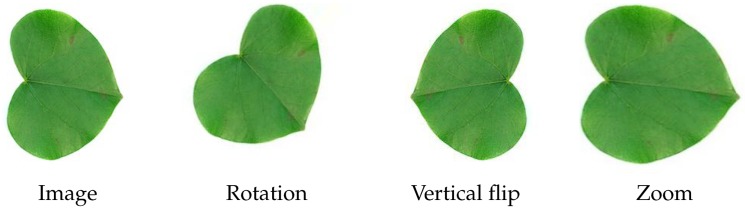
Transformations examples.

Additionally, during the training phase we randomly apply some affine transformations to input images. Transformations applied include rotation (Integer, [0,360]), vertical flip (Boolean), horizontal flip (Boolean), zoom (Float, [−10,10]), shift in the *x* direction (Integer, [−10,10]), shift in the *y* direction (Integer, [−10,10]). For each transformation, the parameter value is chosen randomly in the indicated interval. We use a probability of p=0.5 to decide whether or not we apply any of the above transformations. Figure 7 shows the application of some of the transformations on an image.
**Algorithm 1:** Building the batches of 3-tuples for CSN training.
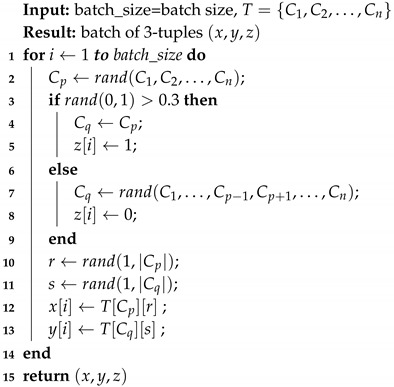


During validation phase, the CSN is validated periodically by means of a batches strategy similar to the one used during the training phase except that we do not apply affine transformations to the input images. Algorithm 2 describes the validation procedure used. Here, y_true is a batch of 3-tuples generated by the procedure described in Algorithm 1, y_pred is the prediction made by the CSN for the batch y_true, and tol is a threshold that must be determined experimentally, because it depends on the domain of application. In our case we use tol=0.5.
**Algorithm 2:** Validation procedure.
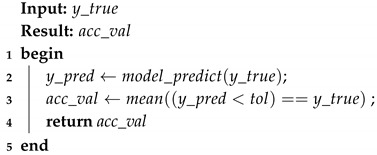


#### 3.4.3. Plant Species Classification Strategy

Once the CSN has been fine-tuned, we can identify the species of a new specimen picture by comparing its extracted features vector with the features vector of a reference set stored for each of the species. The species closest to the specimen is accepted and all other species are rejected when top-1 accuracy is measured and the three closest species are accepted when top-3 accuracy is measured.

Given a new image *q*, the class that is assigned to *q* is the class of the reference set that is closest to *q*. A reference set is composed of images of the same class, which are used to calculate an average similarity with image *q*. So, the class of image *q* is the class of the reference set that on average is more similar to *q*.

More formally, let us say we have *n* classes and that the $C_{i}$, with i=1,2,…,n, are the *n* reference sets, each with $|C_{i}|$ images. To classify a new image *q* in one of the *n* classes available, we calculate the average similarity of image *q* to each of the reference sets $C_{i}$, using the similarity function *d* learned by the convolutional Siamese network, as follows

(3) $${\bar{S}}_{i}\left(q,C_{i}\right)=\frac{1}{|C_{i}|}\sum_{x_{i}\in C_{i}}d\left(q,x_{i}\right)\;i=1,2,\ldots ,n$$

Then, the class $C^{⋆}$ predicted for image *q* is given by

(4) $$C^{⋆}=\mathrm{argmin}\left({\bar{S}}_{1}\left(q,C_{1}\right),{\bar{S}}_{2}\left(q,C_{2}\right),\ldots ,{\bar{S}}_{n}\left(q,C_{n}\right)\right)$$

**Figure 8:**
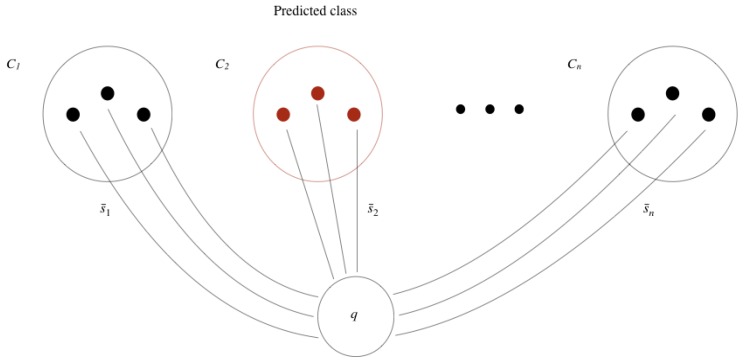
Classification of a new image *q*, given *n* reference sets, each with three images.

That is, the predicted class for image *q* is the class that on average is more similar to *q*, according to the similarity function *d* and reference sets $C_{i}$. Figure 8 illustrates the process of classification of a new image *q*, where the predicted class is $C_{2}$ and each reference set has three images.

### 3.5. Experiments

All experiments were conducted on a desktop computer with an Nvidia GeForce GTX 1070 GPU with 8GB GDDR5 of memory and a Ryzen 7 2700X AMD CPU with 32 GB of memory.

We train the CSN with 500 epochs using the Adam optimizer with initial learning rate of lr=0.00001, $\beta_{1}=0.9$ and $\beta_{2}=0.999$. The CNN was trained with 2000 epochs using the Adam optimizer with the same parameters of the CSN. Both models were implemented with Keras [27] using Tensorflow 2.0 as backend.

In the loss function defined by Equation (2), we have to specify a margin *m* to optimize the proposed CSN. This value was chosen experimentally, by training the network during 500 epochs for different values of *m*. Table 1 shows one of the results obtained when using the training and validation sets of experiment $E_{10}$. Consequently, to train the proposed CSN network, a suitable value for the margin could be m=1.

#### 3.5.1. Scenario 1

In this scenario we conduct the following experiments: $E_{i}$ for *i* in {5,10,15,20,25,30}. In each experiment $E_{i}$, both the CSN and the CNN are trained and validated from scratch with datasets that comprise *i* pictures per species, extracted from the Flavia dataset. Then, each trained model is tested with another, constant dataset Test that comprises 320 images (10 images per species) also from Flavia. The obtained accuracy is compared with the results of feeding the same training, validation, and testing datasets to a CNN in order to determine if there is a threshold value *t* for dataset size that defines the intervals for which either the CSN or the CNN has better accuracy. In addition to experiments $E_{i}$, for *i* in {5,10,15,20,25,30}, we also conduct an experiment, which we simply call *E*, that uses all images in Flavia except those in Test. The number of images per species in Flavia is in the 40 to 67 range.

#### 3.5.2. Scenario 2

The aim of the second experiment is to evaluate the discriminatory power of a CSN when trying to identify images of species unseen before, i.e., not used in the training phase. This provides a measure of how scalable the model is when moving from Scenario 1 to Scenario 2. This is a transfer learning scenario. For this purpose, we randomly choose 20 species of Costa Rican trees from the CRleaves dataset. For each of these species we simulate that we have only a “few images”. Thus, we randomly select 3 reference images and 3 testing images. Consequently, the total number of species considered in the testing phase is 52, that is, 32 species from the Flavia dataset plus 20 species from the CRleaves dataset (unseen during the training phase). For both the CSN and the CNN, we measure the top-1 and top-3 accuracy for each of the 52 species, the average top-1 and top-3 over the 52 species, and we are particularly interested in the average top-1 and top-3 accuracy over the new 20 Costa Rican species.

Finally, because the experiments in Scenario 1 generate seven models of CSNs and CNNs (one for each experiment $E_{i}$ plus the model for experiment *E*), we need to select one model for this transfer learning experiment. Given that those experiments identify an approximate value of threshold *t*, we choose the models for the CSN and CNN generated by experiment $E_{i}$, such that i≤t and *i* is the highest value for which the CSN model has an average accuracy equal or better than the CNN model.

## 4. Analysis of Results

To determine the threshold value, we compare the accuracy of the CSN with the accuracy achieved by the CNN, using in both cases the same datasets for training, validation, and testing.

The top-1 and top-3 accuracy of the CSN model are calculated by using Equations (3) and (4). In each experiment $E_{i}$, *i* in {5,10,15,20,25,30}, we use, as reference sets, the 32 classes with *i* elements used during the corresponding training phase. The top-1 and top-3 accuracy of the CNN model were calculated as usual. Table 2 summarizes the top-1 and top-3 accuracy obtained for each model. Set Test was the same for all experiments $E_{i}$.

**Figure 9:**
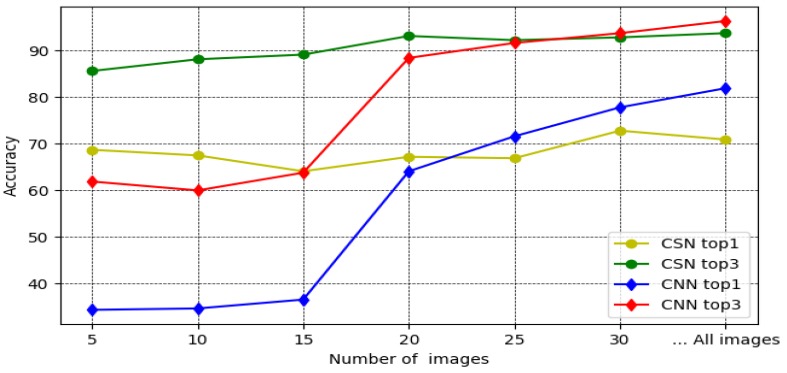
Top-1 and top-3 accuracy for the CSN and CNN.

Figure 9 shows graphically the results of Table 2. We can see that for over 25 images per species, the top-1 and top-3 accuracy of the CNN is better. However, for 20 or less images per species, the top-1 and top-3 accuracy of the CSN is consistently better than that of the CNN. Thus, we can say for the threshold value *t* that 20≤t≤25.

Note that top-3 accuracy of the CSN is between 85.6% and 93.7%, which is acceptable and comparable to the CNN’s top-3 accuracy for 30 images per species. Furthermore, the best performance of the CSN occurs for 30 images per species (experiment $E_{30}$), as it has the best top-1 accuracy of 72.8% and the top-3 accuracy of 92.8%.

As indicated in Section 3.5.2, our second scenario evaluates the CSN’s and CNN’s ability to identify images of classes unseen before. Given that we obtained that 20≤t≤25, this experiment is conducted by using the models generated in experiment $E_{20}$.

We retrained the CNN with the training set used in experiment $E_{20}$ plus the new 20 Costa Rican tree species, each of which has 3 reference images, distributed as follows: 2 for training and 1 for validation.

**Figure 10:**
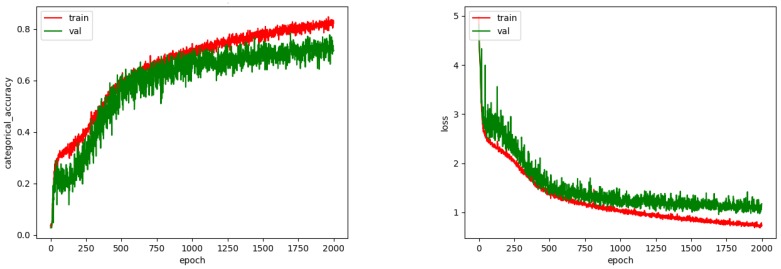
Accuracy and loss for CNN.

The CNN was trained with 2000 epochs and the best weights were saved for the testing phase. Figure 10 shows the accuracy and loss achieved during training. As we can see, after 1500 epochs an overfitting starts to show, perhaps due to the small amount of images.

The top-1 and top-3 accuracy achieved by the CNN with these 52 species are 62.3% and 82.3%, respectively. This is lower than those obtained in experiment $E_{20}$ (see Table 2). This was expected because the CNN was retrained and validated with just 3 images for the 20 new species from Costa Rica. Additionally, the top-1 and top-3 accuracy achieved by the CSN with the 52 species were 60.8% and 85.4%, respectively. Both of them are also lower than the respective accuracies obtained in experiment $E_{20}$ but without any retraining.

Now, if we compare the results of the CNN with the CSN, they are roughly equivalent. This is not surprising, considering that in the experiment $E_{20}$ they were already similar in terms of accuracy. Thus, for this dataset with 52 species, it does not make much difference if we use the CSN or the CNN. Nevertheless, in a scenario, where we cannot retrain the networks, clearly the CSN would be preferable because the CNN would fail for the new 20 species.

Table 3 summarizes the top-1 and top-3 accuracy obtained by both the CSN and the CNN for the 20 new species. Higher accuracies are shown in boldface. We can see that, overall, the average top-1 accuracy of the CSN is 8% better than the CNN. In addition, the top-3 accuracy is also better by 11%. Thus, the CSN is a better alternative to identify species unseen during the training phase, even if the CNN is retrained.

**Figure 11:**
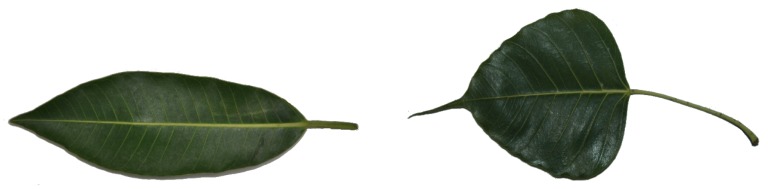
*Ficus religiosa* and *Ficus insipida* images.

At the individual species level, the CSN performs better in a small majority of cases. The top-1 accuracy of the CSN is better than the top-1 accuracy of the CNN in 9 cases and worse in 4 cases. Additionally, top-3 accuracy of the CSN is better than the top-3 accuracy of the CNN in 10 cases and worse in 6 cases. We can see in Table 3 that one of them, *Plumeria rubra*, was not identified by any of the models, even when top-3 accuracy is measured. This species is depicted in Figure 3, second row, sixth image. Perhaps the shape is very generic and thus hard to distinguish from other species. Additionally, the accuracy of the CSN is very poor for species *Bauhinia purpurea* (see Figure 3, first row, fourth image), but the CNN achieved very good accuracy. Conversely, the species *Ficus insipida* and *Ficus religiosa* are difficult to identify by the CNN but not for CSN. Figure 11 shows an image of these two species.

## 5. Conclusions and Future Work

Perhaps the main obstacle in applying deep learning techniques is the scarcity of labeled data for the adequate training of a CNN model. Data augmentation through geometric transformations or the generation of artificial data are alternatives that have been proposed to tackle this problem but in some cases they are not sufficient. The proposed CSN was successfully applied to small datasets of plant leaf images that have between 5 and 30 images per species. We carried out experiments in which we gradually increased the number of images available per species and evaluated the accuracy achieved by both networks with the same testing set. This led us to conclude that for datasets with less than 20 images per species, the convolutional Siamese network is better than the convolutional network, at least in this domain of application. From this threshold point onwards, the convolutional Siamese network is surpassed by the classical convolutional neural network. The experiments with both networks were carried out under equivalent conditions, which allowed the results to be comparable.

An important advantage of a CSN over a classical CNN is that its discriminatory power can be generalized without any retraining for new species identifications. To test this, we used the CSN that was trained with images of leaves from the Flavia dataset to identify—with quite good levels of average accuracy— plant species from Costa Rica that were not represented in Flavia. Even though the CSN was not retrained with data from the CRleaves dataset and the CNN was, the average top-1 and top-3 accuracy of the CSN was better (56% vs. 48% and 81% vs. 70%, respectively). Besides, the top-1 accuracy of the CSN was also better in the identification of nine individual species from the CRleaves dataset (45%) and inferior for only four of them (20%). For the top-3 accuracy, the CSN was also better in the identification of 10 individual species (50%) from the CRleaves dataset and inferior for six of them (30%). Overall, the CSN-based classifier achieves better top-1 and top-3 accuracy in the subset composed of the 20 new species from Costa Rica, thus it is the best alternative to identify species unseen during the training phase and in a scenario of few data.

Because the scope of this experiment is limited to leaf image datasets, further research is needed to address the same issues when other types of images are used. For example, herbarium sheet datasets have been used to identify plants with CNNs [14,29]; however, for certain regions, datasets are rather small. Additionally, we are currently working on the problem of identifying tree species in a context where very few images are available worldwide, namely, tree species identifications based on wood cut images. Preliminary work on protocols for collecting wood samples, digitizing images, and using CNNs for tree species identification has been published in [30,31]. However, because the number of samples in xylotheques worldwide is relatively small, exploring the use of CSNs seems a promising alternative.

## Figures and Tables

**Table 1:** Choosing the value of *m*.

*m*	0.5	0.75	1	1.25	1.5	1.75	2
Accuracy (CSN)	86	89	93	92	91	85	80

**Table 2:** Scenario 1: accuracy achieved for different dataset sizes.

Experiment	Dataset Size			CSN		CNN	
	Training	Validation	Testing	Top-1	Top-3	Top-1	Top-3
$E_{5}$	4	1	10	68.7	85.6	34.4	61.6
$E_{10}$	8	2	10	67.5	88.1	34.7	60.0
$E_{15}$	12	3	10	64.1	89.1	36.6	63.8
$E_{20}$	16	4	10	67.2	93.1	64.1	88.7
$E_{25}$	20	5	10	66.9	92.2	71.6	91.6
$E_{30}$	24	6	10	72.8	92.8	77.8	93.7
*E*	[32,54]	[8,13]	10	70.9	93.7	81.9	96.3

**Table 3:** Experiment 2: accuracy/species for 20 Costa Rican tree species.

Species	CSN		CNN	
	Top-1	Top-3	Top-1	Top-3
*Passiflora platyloba*	**60**	60	40	**100**
*Plumeria rubra*	0	0	0	0
*Ficus insipida*	**40**	**100**	20	20
*Calea urticifolia*	20	40	20	**80**
*Hymenaea courbaril*	**100**	**100**	40	80
*Bombacopsis quinata*	**60**	60	40	**80**
*Astronium graveolens*	40	80	40	**100**
*Blakea maurofernandeziana*	60	**100**	60	60
*Allophylus psilospermus*	**80**	**100**	60	80
*Artocarpus heterophyllus*	20	**100**	**40**	40
*Cochlospermum vitifolium*	**80**	100	60	100
*Bauhinia purpurea*	0	20	**80**	**80**
*Malachra alceifolia*	**100**	**100**	20	40
*Aristolochia leuconeura*	60	80	**80**	**100**
*Plectranthus amboinicus*	60	**80**	60	60
*Cajanus cajan*	100	100	100	100
*Crescentia alata*	0	100	**100**	100
*Anacardium excelsum*	40	**100**	40	60
*Ficus religiosa*	**100**	**100**	0	40
*Schinus terebinthifolius*	**100**	**100**	60	80
**Average accuracy**	**56**	**81**	48	70
